# Prevention of the Toxic Effects of Cocaine

**Published:** 1890-08-09

**Authors:** 


### PREVENTION OF THE TOXIC EFFECTS OF COCAINE.

The addition of a small quantity of phenol to solutions of cocaine has already been proposed with a view to preventing the growth of moulds, which not only break up the alkaloid but render the solution pungent and irritating. Dr.Gluck however maintains that it also obviates the toxic effects,averts congestive reaction, and increases the anæsthetic action. That it preserves the solution from decomposition by fungoid organisms is no doubt true, but it is not easy to conceive how it can prevent the unpleasant effects on the cerebral circulation, which are apparently but an extension of the vascular constriction on which its value as a local application to congested mucous membranes depends, and following its too rapid absorption into the circulation as when injected subcutaneously. In such cases, which, so far as we know, have occurred for the most part, if not exclusively, after injections performed in unsuitable parts, as the gums in dentistry, where the skin cannot be raised in a fold, or elsewhere in persons whose abundant subcutaneous fat presents an impediment to the proper introduction of any injections, the drug probably has gained direct access to veins or capillaries, as in corresponding accidents attending the injection of morphia and other alkaloids. A case, indeed, has come under our notice in which symptoms of cocaine poisoning followed its too lavish application to the fauces by means of a large camel's-hair brush, but in that it may have been swallowed.

We make it a rule, which we believe to be a safe one, to employ only known quantities externally to mucous surfaces, or in injections where a fold of skin can be easily raised by the fingers. The only occasion on which we have ourselves observed the least signs of facial and cerebral anæmia was when we deviated from our usual practice by injecting it into the ball of the thumb for the extraction of a needle. The threatened symptoms immediately passed off on the inhalation of Amyl nitrite. This, a true physiological antagonist or antidote, should always be at hand, but how phenol can counteract the danger, or indeed how anything could that did not neutralise its therapeutic action we fail to see. Dr. Gluck states that ordinary solutions become inert in three or four days; but using boiled (sterilized) water we find no impairment of 10 per cent. solutions after a month.

